# Suppression of Cytosolic Phospholipase A2 in the Ventromedial Hypothalamus Induces Hyperphagia and Obesity in Male Mice

**DOI:** 10.3390/ijms26157532

**Published:** 2025-08-04

**Authors:** Takashi Abe, Taiga Ishimoto, Yudai Araki, Ziwei Niu, Changwen Li, Jinxiao He, Samson Ngurari, Chitoku Toda

**Affiliations:** Department of Neuroscience for Metabolic Control, School of Medicine, Kumamoto University, Kumamoto 860-8555, Japan; fortomorrow.1125@gmail.com (T.I.); y.araki0216@gmail.com (Y.A.); ziweiniu98@gmail.com (Z.N.); lichangwen1994@gmail.com (C.L.); shawnleetttt@outlook.com (J.H.); ngurariwain007@gmail.com (S.N.)

**Keywords:** appetite, phospholipid, prostaglandins, body weight, overeating, VMH

## Abstract

We recently reported that phospholipase A2 (PLA2)-mediated production of prostaglandins within the ventromedial hypothalamus (VMH) plays a critical role in systemic glucose homeostasis. However, the role of PLA2 in the VMH in regulating food intake is still unclear. Here, we attempted to investigate the role of PLA2 in regulating food intake and body weight in male mice. We injected an adeno-associated virus encoding short hairpin RNA (AAV-shRNA) targeting cytosolic phospholipase A2 (shPla2g4a) into the VMH. We assessed food intake, body weight, oxygen consumption, glucose tolerance, and insulin sensitivity. Three weeks after the AAV injection, the shPla2g4a group exhibited increased food intake and body weight gain compared to controls (shSCRM). Energy expenditure, oxygen consumption, and respiratory quotient (RQ) were comparable between groups. Our findings suggest that the cPLA2-mediated pathway in the VMH is critical for feeding behavior and maintaining energy homeostasis. Further investigation is needed to elucidate the underlying mechanisms.

## 1. Introduction

Prostaglandins (PGs) are widely distributed in the central nervous system (CNS) and play diverse physiological functions. [1]. PGs regulate not only inflammation but also appetite, sleep-wake, and emotion [1,2,3,4,5,6]. Cytosolic phospholipase A2 (cPLA2) catalyzes phospholipids containing arachidonic acid as a precursor for PG synthesis [7,8]. Recently, we reported that cPLA2-knockdown (KD) in the ventromedial hypothalamus (VMH) steroidogenic factor-1 (SF-1) neuron of mice fed a regular chow diet (RCD) leads to decreased insulin sensitivity in skeletal muscle [9]. This finding suggests that cPLA2 enhances systemic insulin sensitivity by activation of dmVMH glucose-excited (GE) neurons. Additionally, another study demonstrated that inhibition of PG synthesis by cyclooxygenase-1/2 (COX-1/2) inhibitors impairs the counter-regulatory response by suppressing glucagon sensitivity [10]. In cPLA2-KD mice, cFos expression is specifically suppressed in the dorsomedial VMH (dmVMH). In contrast, in wild-type mice, intraperitoneal (i.p.) glucose administration increases cFos expression in the VMH and the arcuate nucleus (ARC) [9]. These studies suggest that ARC neurons are regulated by PG, and thus, it is required to evaluate the function of PG-regulated neuronal activity in the hypothalamus on energy homeostasis.

The regulation of the CNS in the termination of feeding after a meal is complex and involves multiple mechanisms. The extracellular signal-regulated kinase (ERK) in the hypothalamus mediates the anorexigenic and thermogenic effects of leptin, suppressing food intake and increasing sympathetic outflow to brown adipose tissue [11]. ERK in the nucleus tractus solitarius (NTS), activated by the gut-derived satiety signal cholecystokinin (CCK), also plays a crucial role in suppressing food intake [12]. The melanocortin receptor (MC4R) agonist MTII also increases p-ERK in the NTS, suppressing food intake [13]. Cyclic AMP (cAMP)-response element binding protein (CREB) is a transcription factor that is regulated by ERK. In the NTS, the cAMP-ERK-CREB cascade may serve as a molecular integrator for converging satiety signals from the gut and adiposity signals from the hypothalamus [14]. CREB targets COX-2 expression, which is crucial for PG synthesis [15]. However, the relationship between insulin-induced ERK signaling and PG synthesis, particularly in regulating hyperphagia, remains unclear. This study aimed to investigate the role of hypothalamic PG in the development of hyperphagia. We found that cPLA2-KD in the hypothalamus of RCD-fed wild-type male mice resulted in a significant increase in food intake, leading to a body weight gain of approximately 5 g within three weeks. This study demonstrated that hypothalamic neuronal activity regulated by cPLA2 and its downstream factors plays a critical role in controlling feeding behavior and obesity.

## 2. Results

**Figure 1:**
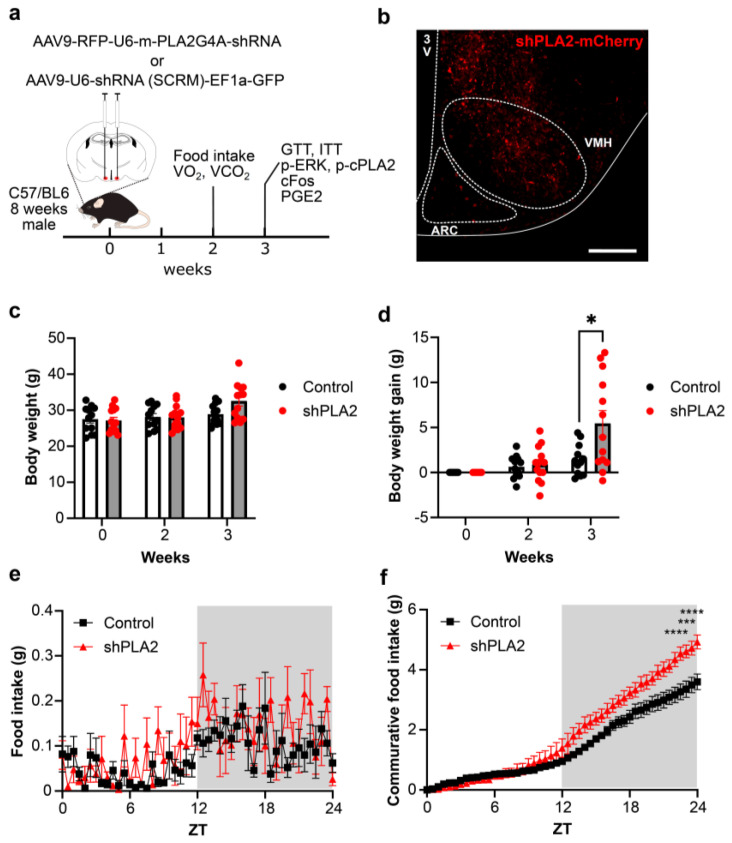
shRNA against cPLA2 in the hypothalamus increased body weight and food intake in male mice. (**a**) A schematic illustrating the experimental timeline of AAV injection into the VMH, food intake, oxygen consumption, tolerance tests, and immunohistochemistry. (**b**) Representative image of mCherry expression after AAV injection. (**c**) Body weight in 0, 2, and 3 weeks after the injection of shRNA against cPLA2 into the VMH. (**d**) Body weight gain in 0, 2 and 3 weeks after the shSCRM (*n* = 7) or shPLA2 (*n* = 7) injection. (**e**) Food intake in 2 weeks after the shSCRM (*n* = 5) or shPLA2 iniection (*n* = 5). (**f**) Cumulative food intake (ZT 0–24). Scale bar: 200 µm. All data represent the mean ± SEM; * *p* < 0.05; *** *p* < 0.001; **** *p* < 0.0001. One-way ANOVA followed by Tukey’s multiple comparison tests and two-way ANOVA followed by Sidak multiple comparison tests were used for statistical analysis.

To knock down PLA2g4a gene expression, we injected AAV9-RFP-U6-m-PLA2G4A-shRNA or AAV9-U6-shRNA (SCRM)-EF1a-GFP bilaterally in the VMH of C57BL mice (Figure 1a). mCherry or GFP derived from viral vectors was found in both the VMH and ARC, suggesting that AAV successfully infected the hypothalamus (Figure 1b). shRNA against cPLA2 decreased cPla2 mRNA expression in the hypothalamus to less than 50% of that in control animals [10]. The body weight of shPLA2 mice was comparable to that of shSCRM mice at two weeks after AAV injection, but it tended to increase at three weeks post-AAV injection (Figure 1c). Importantly, when we calculated weight gain, shPLA2 mice showed significantly increased weight gain compared to shSCRM mice at three weeks (Figure 1d). This obesity phenotype persisted for at least six months with mice reaching 60 g in body weight. Furthermore, food intake was significantly higher in shPLA2 mice at two weeks post-AAV injection (Figure 1e,f).

**Figure 2:**
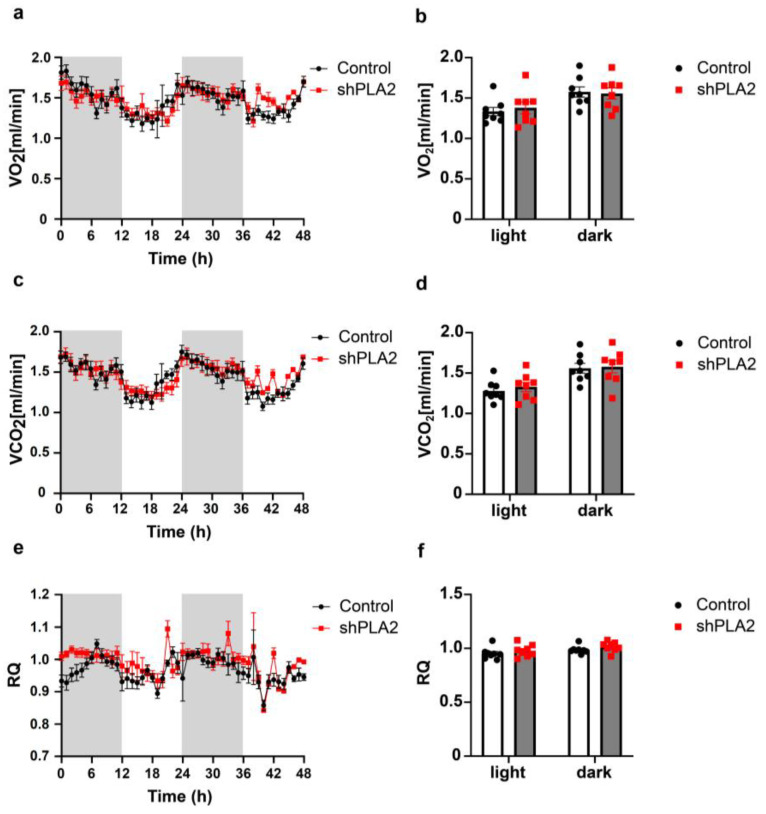
shRNA against cPLA2 in the hypothalamus did not affect oxygen consumption in male mice. (**a**) Volume of oxyoen consumpton (VO_2_). (**b**) Average VO_2_ in light (0–6 and 24–36 h) and dark (12–24 and 36–48 h) period. (**c**) Volume of carbon dioxide producton (VCO_2_). (**d**) Average VCO_2_ in light and dark period. (**e**) Respiratory quotient (RQ). (**f**) Average RQ in light and dark period. All data represent the mean ± SEM; no significant dfferences at *p* < 0.05 The unpaired Student’s *t*-test (two-tailed *p*-value) and two-way ANOVA followed by Sidak multiple comparison tests were used for statistical analysis.

**Figure 3:**
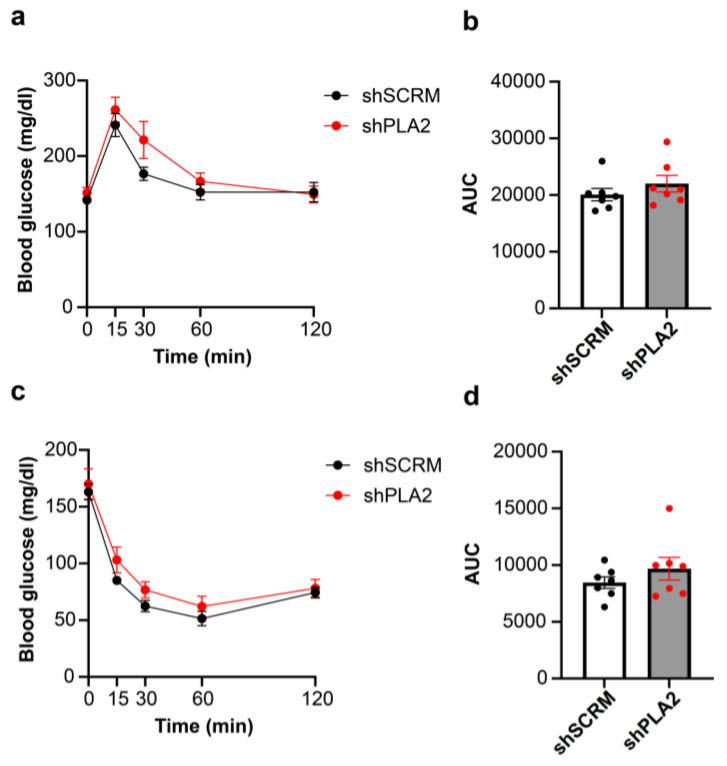
Glucose tolerance test and insulin tolerance test in shSCRM- or shPLA2-injected male mice. (**a**) Blood glucose levels (0–120 min) during i.p. glucose tolerance test (GTT) three weeks after the injection of shSCRM or shPLA2 into the VMH of male mice. (**b**) Area under the curve (AUC) of blood glucose levels during i.p. GTT. (**c**) Blood glucose levels (0–120 min) during i.p. 0.75 U/kg insulin tolerance test (ITT) three weeks after the injection of shSCRM (*n* = 7) or shPLA2 (*n* = 7) into the VMH. (**d**) Area under the curve (AUC) of blood glucose levels during i.p. ITT. All data represent the mean ± SEM; no significant differences at *p* < 0.05. The unpaired Student’s *t*-test (two-tailed *p*-value) and two-way ANOVA followed by Sidak multiple comparison tests were used for statistcal analysis.

We next examined oxygen consumption, carbon dioxide production, and the respiratory quotient (RQ) using an in vivo calorimetry system. However, these parameters did not differ between the shPLA2 and control groups (Figure 2a–f). In addition, hypothalamic shPLA2 did not affect glucose tolerance or insulin sensitivity at three weeks post-AAV injection (Figure 3a,b).

**Figure 4:**
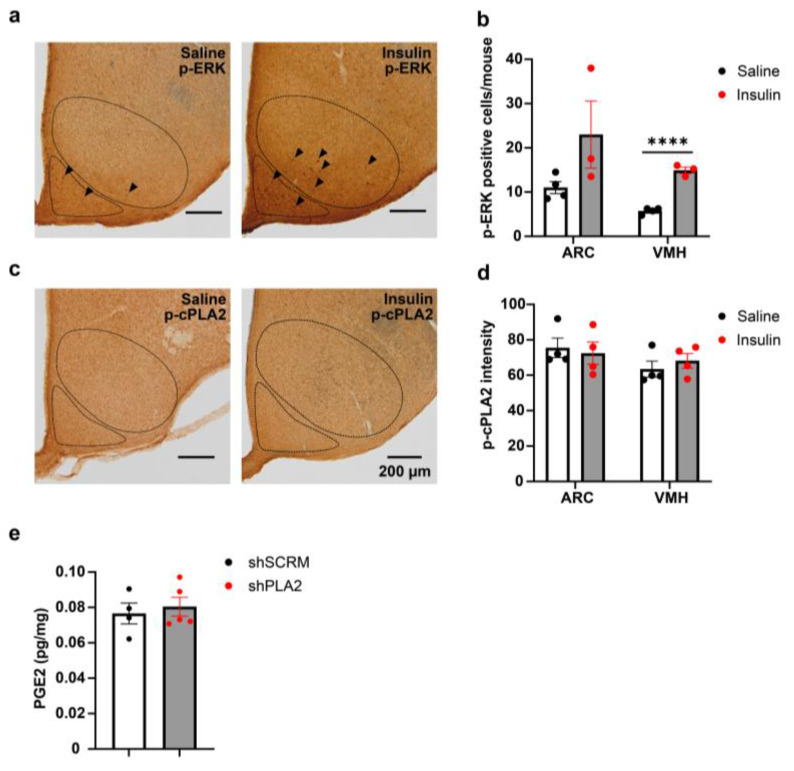
Insulin injection increased phospho-ERK expression but not phospho-cPLA2 expression in male mice. (**a**) Representative images of phospho-ERK (p-ERK) immunohistochemistry in the hypothalamus 60 min after i.p. injecton of saline (*n* = 4) or 0.75 U/kg insulin (*n* = 3). (**b**) Quantification of p-ERK expression in the hypothalamus; ARC: arcuate nucleus; VMH: ventromedial hypothalamus. (**c**) Representative images of phospho-cPLA2 (p-cPLA2) immunohistochemistry in the hypothalamus 60 min after i.p. injection of saline (*n* = 4) or 0.75 U/kg insulin (*n* = 4). (**d**) Quantification of p-ePLA2 expression in the hypothalamus. (**e**) Prostaglandin E2 levels in the hypothalamus of shSCRM (*n* = 4) or shPLA2 (*n* = 5) injected mice. Arrowhead: p-ERK immunoreactivity. Scale bars: 200 µm. All data represent the mean ± SEM; **** *p* < 0.0001. The unpaired Student’s *t*-test (two-tailed *p*-value) was used for statistical analysis.

To evaluate the effect of insulin on the neuronal activation modulated by ERK signaling, we performed immunohistochemical staining for phosphorylated p44/42 MAPK (p-ERK1/2) in the hypothalamus following insulin injection (Figure 4a). Sixty minutes after insulin i.p. administration, p-Erk expression in the VMH was significantly higher than in the saline-treated control group (Figure 4b). In contrast, while p-Erk expression in the ARC indicated a trend toward an increase, the difference was not statistically significant (Figure 4b). Given that insulin administration enhances PG production in the hypothalamus in our recent finding [10], we also performed immunohistochemical staining for phosphorylated cPLA2 (p-cPLA2) in the hypothalamus of these mice (Figure 4c). Although the mean intensity of p-cPLA2 staining tended to increase after insulin injection in the VMH and ARC, no significant differences were observed between groups (Figure 4d). To evaluate the effect of cPLA2-KD on PG synthesis, we measured prostaglandin E2 (PGE2) levels by ELISA. PGE2 in the hypothalamus was also comparable between shSCRM and shPLA2 injected mice (Figure 4e).

**Figure 5:**
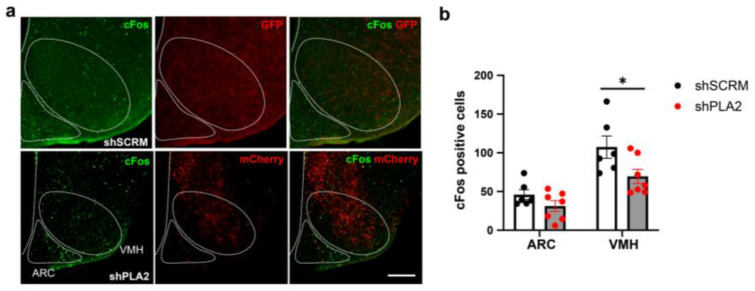
shRNA against cPLA2 in the hypothalamus decreased cFos expression after the insulin injection in male mice. (**a**) Representative imapes of cFos immunohistochemistry ih the hypothalamus 60 min after i.p. injection of 0.75 U/kg insulin. GFP was replaced with red using ImageJ. (**b**) Quantification of cFos expression in the hypothalamus of shSCRM (*n* = 6) or shPLA2 (*n* = 7) injected mice. Scale bar: 200 µm. All data represent the mean ± SEM; * *p* < 0.05. The unpaired Student’s *t*-test (two-tailed *p*-value) was used for statisical analysis.

We also analyzed the effect of cPLA2-KD on the insulin-induced cFos expression in the hypothalamic nuclei. cFos immunohistochemical staining was performed in the hypothalamus 60 min after insulin administration in both the shPLA2 and shSCRM groups (Figure 5a). cFos expression was significantly reduced in the VMH of shPLA2 mice (Figure 5b). cFos expression in the ARC tended to decrease in shPLA2 mice, but it was not significant (Figure 5b).

## 3. Discussion

In the present study, suppressing hypothalamic cPLA2 via shRNA in male mice resulted in increased food intake and weight gain. During this period, key metabolic parameters like oxygen consumption (VO_2_ or VCO_2_), respiratory quotient (RQ), glucose tolerance, and insulin tolerance remained unchanged after hypothalamic shPLA2 injection. Notably, insulin-induced cFos expression in the VMH was decreased by shPLA2. These findings collectively suggest that PLA2-mediated neuronal activity in the VMH is crucial for appetite regulation and body weight control, and insulin has a key role in these regulations.

Numerous studies have demonstrated the critical role of SF1 neurons in the VMH in regulating feeding behavior. The deletion of leptin receptors in VMH-SF1 neurons leads to diet-induced obesity [16]. The loss of insulin receptors in SF1 neurons suppresses weight gain under a high-fat diet via phosphoinositide 3-kinase (PI3K) signaling [17]. Furthermore, the deletion of PI3 phosphatase in SF1 neurons induces excessive PI3K activation in the VMH, leading to hyperphagia and weight gain even under a regular chow [17]. Additionally, chemogenetic inhibition of SF1 neurons using DREADDs enhances appetite and increases food intake [18]. These studies collectively suggest that hyperphagia and subsequent weight gain result from changes in the activity of VMH SF1 neurons, which are regulated by humoral factors involved in postprandial feeding suppression. Thus, our data of decreased VMH activity and increased weight is consistent with these reports.

Our findings suggest that a reduction in arachidonic acid—the substrate for PG synthesis catalyzed by COX-1/2—may suppress PG-regulated neuronal activity in the hypothalamus. This is supported by our observation that cPLA2-KD in the hypothalamus reduced insulin-induced cFos-positive cells in the VMH. Previous reports establish that CREB regulates COX-2 activity [15,19]. A study showed that SF1 neuron-specific deletion of CRTC1, a co-activator of CREB, leads to hyperphagia in mice fed a high-fat diet [20]. We also recently reported that inhibiting PG production using ibuprofen, a COX inhibitor, suppressed insulin-induced cFos expression in the ventrolateral VMH (vlVMH) [10].

Insulin exerts both direct and indirect effects on food intake regulation, directly suppressing appetite via neuronal pathways [21]. To investigate insulin-induced anorexigenic neuronal functions, we analyzed p-ERK and p-cPLA2 immunoreactivity in the hypothalamus of insulin-treated mice. In the present study, insulin injection increased p-ERK but not p-cPLA2 in the hypothalamus. Insulin is known to activate the ERK-CREB-COX pathway in the hypothalamus [22]. In this study, insulin increased p-ERK without affecting p-cPLA2, suggesting that PG production may occur via the ERK–CREB–COX pathway independently of cPLA2. In addition, arachidonic acid may be supplied by other phospholipases [8]. Such alternative pathways could partially preserve PG synthesis, which may explain why cPLA2-KD did not affect several parameters, such as RQ, glucose tolerance, and insulin sensitivity. VMH neurons projecting to ARC-POMC neurons are essential for suppressing food intake [23]. In line with this, cFos in the ARC tended to decrease in shPLA2 mice, which may reflect reduced activity of POMC neurons. Therefore, our findings suggest that cPLA2-regulated PG synthesis in the VMH is essential for feeding termination via POMC neurons, as its knockdown resulted in hyperphagia and subsequent obesity.

Postprandial hyperinsulinemia also affects cerebrospinal fluid (CSF) insulin levels [24]. In rats, glucose infusion and refeeding resulted in elevated CSF insulin [24]. These findings suggest that peripheral insulin can reach the CNS and regulate food intake and body weight. Insulin treatment increases ERK1/2 expression in hypothalamic cells in vitro [22]. Short-term fasting also increases p-ERK1/2 levels in the hypothalamus, partially mediated by centrally produced insulin [25]. Enhanced ERK1/2 activity, achieved through deletion of its phosphatases DUSP6/8, leads to resistance to diet-induced obesity, improved glucose tolerance, and reduced serum triglycerides and lipid content in the liver and visceral adipose tissues [26]. In hypothalamic neurons, ERK1/2 mediates glucose-regulated POMC gene expression, influencing metabolic homeostasis [27]. These studies indicate that ERK activation by diet, blood glucose, and insulin is involved in both the improvement and worsening of metabolic-related diseases. Future research will need to examine hypothalamic p-ERK-positive neuron types to elucidate their relationship with feeding suppression.

Previous research indicates that cPLA2-controlled PG synthesis plays a role in the activity of both glucose-excited neurons in the dorsomedial VMH (dmVMH) [9] and glucose-inhibited neurons in the ventrolateral VMH (vlVMH) [10]. In rats, LPS-induced anorexia, which is linked to increased expression of corticotropin-releasing factor and α-melanocyte-stimulating hormone in the ARC, is partially reduced by indomethacin, a PG synthesis inhibitor [28]. These findings suggest that PGs regulate the appetite-suppressing neuronal activity in ARC-POMC neurons. Therefore, it is possible that the shPLA2 in our study led to increased food intake by disrupting this pathway.

PGE2 and related inflammatory pathways suppress appetite and limit body weight gain during fever in rodent models. Notably, i.c.v. injections of PGE2 and microinjections into the paraventricular nucleus of the hypothalamus rapidly induce anorexia in rats [29]. Anorexia during inflammation and infection induced by interleukin-1 is known to be mediated by PGE2 [30]. It has been reported that IL-1-induced anorexia is abolished in microsomal PGE2 synthase-1 knockout mice [31]. COX-2 inhibition also preserves food intake in mice [32]. These findings support that PGE2 mechanisms are associated with decreased appetite and reduced body weight during febrile responses. Given that cPLA2 is essential for arachidonic acid release and subsequent PG synthesis, the hyperphagia observed in shPLA2 mice is consistent with PG-mediated appetite suppression, particularly under stimulated conditions—such as hyperglycemia, postprandial insulin signaling, or satiety—where PGE2 is normally upregulated to limit food intake [9,10]. Further investigation into these mechanisms may provide a potential approach for suppressing hyperphagia and obesity.

PGs play a crucial role in metabolic control and appetite regulation. cPLA2 is crucial in generating arachidonic acid from phospholipids for PG synthesis [33]. In the CNS, PGs have diverse effects on food intake. Centrally administered PGE2 suppresses food intake via the EP4 receptor, whereas PGD2 increases food intake through the DP1 receptor, which is coupled to the Y1 receptor of neuropeptide Y [6]. Our previous study also highlighted that cPLA2-mediated hypothalamic phospholipid metabolism is critical for controlling systemic glucose metabolism under a regular chow diet [9]. In the present study, baseline PGE2 levels were comparable between cPLA2-KD and control. The measured values were markedly lower than those reported in previous studies: 500 nmol/g (approximately 0.176 µg/mg) of brain tissue [34] and 100 pg/mL in CSF [35,36]. Taken together, these findings suggest that PG production under unstimulated conditions is minimal, and that the effect of cPLA2-KD may become apparent under stimulated conditions such as fever, hyperglycemia after a meal, and insulin-induced hypoglycemia. Alternatively, cPLA2-KD may preferentially affect other PGs, such as PGF2α, rather than PGE2 under baseline conditions. Previous studies have shown that PGE2 plays a key role in appetite regulation [29,30,31,32]. Whether cPLA2-KD alters PG production under stimulated conditions, such as feeding or acute insulin administration, remains to be clarified in future studies.

The limitations of this study are that the potential effects of hypothalamic cPLA2-KD on neuronal circuits and neuron types have not been fully assessed. Additionally, since this study exclusively used male mice and AAV to induce knockdown, the potential for sex-specific differences in the effects of cPLA2-KD was not explored. This sex bias limits the generalizability of the findings. Another important consideration for future research is the use of whole-body cPLA2 knockout mice with cPLA2 rescued specifically in the ventromedial hypothalamus. Such an experimental design would help to definitively pinpoint the localized effects of hypothalamic cPLA2 on feeding behavior, distinguishing them from potential systemic influences. It is also difficult to directly apply the findings to humans in a clinical context. These issues should be addressed in future studies to clarify the precise neural mechanisms.

In this study, we demonstrated that hypothalamic cPLA2-KD induces obesity in a short period through increased food intake. During this process, cPLA2-KD suppressed the release of arachidonic acid, a substrate for PG production regulated by the ERK-CREB-COX pathway during postprandial hyperinsulinemia. Since obesity in this study resulted solely from increased food intake without peripheral metabolic changes, it is possible that feeding suppression, mediated by the activation of ARC-POMC neurons and regulated by PGs, was impaired. These findings, particularly the hyperphagia observed in hypothalamic cPLA2-KD, suggest a crucial role for cPLA2-mediated PG synthesis in the suppression of food intake. While PGs, such as PGE2, are well-established mediators of anorexia during inflammatory or febrile states, our study demonstrates that suppression of cPLA2-mediated PG synthesis leads to hyperphagia in normally fed conditions. This highlights a previously underrecognized role for the cPLA2-PGs pathway as a key regulator that prevents excessive food intake and maintains metabolic balance under normal feeding conditions. Although further studies are needed, this study advances knowledge in the regulation of systemic metabolism mediated by the PG synthesis pathway. This finding provides a novel therapeutic target for obesity treatment.

## 4. Materials and Methods

### 4.1. Animals

C57BL/6N male mice (Japan SLC, Shizuoka, Japan) were housed at room temperature with a 12 h light and 12 h dark cycle. The mice had free access to water and a regular chow diet (CLEA Japan, Tokyo, Japan). Experiments were conducted in the Experimental Animal Facility at Kumamoto University (A2024-009). Mice cages were changed once a week, and mouse care was performed according to the guidelines of the Animal Care and Use Committee at Kumamoto University.

### 4.2. Cannula Implantation and Adeno-Associated Virus (AAV) Injection

C57BL male mice were anesthetized with a combination of anesthetics (0.3 mg/kg medetomidine, 4.0 mg/kg midazolam, and 5.0 mg/kg butorphanol). The mice were placed on a stereotaxic instrument (Narishige, Tokyo, Japan). A hole in the skull was opened with a dental drill. A stainless-steel cannula (Plastics One, P1 Technologies, Roanoke, VA, USA) was inserted into the VMH using the following coordinates: anterior-posterior (AP) direction: −1.5 (1.5 mm posterior to the bregma); lateral (L): ±0.4 (0.4 mm lateral to the bregma); and dorsal-ventral (DV): −5.7 (5.7 mm below the bregma on the surface of the skull). For intracerebroventricular (i.c.v.) injection, the coordinates are as follows: AP: −0.3, L: 1.0, DV: −2.0. Cannulae were secured on the skulls with cyanoacrylic glue, and the exposed skulls were covered with dental cement. For AAV injection, the mice were injected in both sides of the VMH with a maximum of 0.3 µL AAV9-RFP-U6-m-PLA2G4A-shRNA (1.0 × 10^12^ GC/mL, shAAV-268768, Vector Biolabs, Malvern, PA, USA) or AAV9-U6-shRNA (scrumble, SCRM)-EF1a-GFP (1.0 × 10^12^ GC/mL, SL100894, Signagen Laboratories, Frederick, MD, USA) using the following coordinates: AP: −1.5, L: ± 0.5, and DV: −5.7. Open wounds were sutured after the viral injection. The mice were allowed to recover for 5–7 days before the experiments began.

### 4.3. Body Weight Gain, Food Intake, and Oxygen Consumption

To evaluate the effects of hypothalamic cPLA2-KD on body weight and food intake, we compared food intake between the control and shPLA2 groups two weeks after AAV administration. Mice were placed in food intake measurement cages (cFDM-300AS, Melquest, Toyama, Japan) and habituated for 24 h, followed by food intake measurement from ZT 0 to 24. Indirect calorimetry data from mice injected with shPLA2 or shSCRM were measured using an indirect calorimetry system for 48 h (MK-5000RQ/MS, Muromachi Kikai, Fukuoka, Japan). Mice were acclimated to the measurement chambers for one day prior to data collection.

### 4.4. Prostaglandin E2 ELISA

PGE2 levels in the hypothalamus were measured in basal conditions in mice that received shSCRM or shPLA2 injection into VMH. Mice received shRNA injections five weeks prior to sacrifice. They were euthanized by cervical dislocation, and approximately 10 mg of hypothalamic tissue was collected and immediately frozen on dry ice. Tissue PGE2 levels were determined by ELISA (PGE2 ELISA Kit, Elabscience, Houston, TX, USA), with absorbance measured at 450 nm using a microplate reader (SH9000Lab, Corona Electric, Hitachinaka, Japan).

### 4.5. p-ERK and p-cPLA2 Immunohistochemistry

C57BL/6 male mice received an i.p. injection of saline or insulin (0.75 U/kg). The mice were deeply anesthetized with isoflurane and perfused with heparinized saline, followed by 4% paraformaldehyde transcardially 60 min after the i.p. injection. Brain sections (50 µm each) containing both VMH and ARC were collected. The floating sections were incubated in 1% H_2_O_2_ in 0.1 M phosphate buffer (PB) for 15 min at room temperature (RT) to inhibit endogenous peroxidase activity. After rinsing with PB, the sections were incubated with rabbit-anti-pErk antibody (1:1000, Cell Signaling Technology, #4370S, Danvers, MA, USA) or rabbit-anti-pCpla2 antibody (1:1000, Cell Signaling Technology, #2831S, Danvers, MA, USA) in blocking solution (0.1 M PB containing 4% normal horse serum, 0.1% glycine, and 0.2% Triton X-100) over the weekend at room temperature. After rinsing with PB, the sections were incubated in a secondary antibody (1:500, Biotinylated goat anti-rabbit IgG, Vector Laboratories, BA-1000, Newark, CA, USA) for two hours at room temperature. After rinsing with PB, sections were incubated with ABC solution (VECTASTAIN Elite ABC kit, Peroxidase, Vector Laboratories, CA, USA) for 2 h at room temperature. The sections were rinsed with PB and incubated with DAB solution (DAB Tablet, Wako, Japan) for 20 min at room temperature. The stained sections were rinsed with PB and mounted using Mount-Quick (Daido Sangyo, Toda, Japan). Cells were automatically counted using the ImageJ software (Ver. 2.16.0/1.54p) plugin (ImageJ software plugin, Analyze Particles).

### 4.6. cFos Immunohistochemistry

C57BL/6 male mice received shPLA2 or shSCRM injections 4 weeks before the i.p. injection of insulin (0.75 U/kg). The mice were deeply anesthetized with isoflurane and perfused with heparinized saline transcardially 60 min after insulin injection. Brain sections (50 µm each) containing both VMH and ARC were collected. After rinsing with PB, the sections were incubated with rabbit-anti-cFos antibody (1:1000, Cell Signaling Technology, #2250S, Danvers, MA, USA) in blocking solution (0.1 M PB containing 4% normal horse serum, 0.1% glycine, and 0.2% Triton X-100) overnight at room temperature. After rinsing with PB, the sections were incubated in a secondary antibody (1:500, Alexa 488 secondary antibody, Invitrogen, A11008, Waltham, MA, USA) or (1:500, Alexa 594 secondary antibody, Cell Signaling Technology, 8889S, Danvers, MA, USA) for two hours at room temperature. The stained sections were washed three times with PB and then mounted on glass slides using DAPI Fluoromount-G (Southern Biotech, Birmingham, AL, USA). Cells were automatically counted using ImageJ.

### 4.7. Statistical Analysis

For repeated-measures analysis, a two-way ANOVA was used to analyze values over different time points, followed by the Sidak multiple comparisons test. For the statistical analysis of multiple independent groups, one-way ANOVA was followed by Tukey’s multiple comparisons test. When only two groups were analyzed, statistical significance was determined by the unpaired Student’s *t*-test (two-tailed *p*-value). Prism 10 software (GraphPad Software, San Diego, CA, USA) was used for these statistical analyses. A value of *p* < 0.05 was considered statistically significant. All data are shown as mean ± SEM.

## 5. Conclusions

This study revealed that cPLA2-KD in the VMH induces obesity solely through increased food intake. cPLA2-KD suppressed the production of eicosanoids from membrane phospholipids and inhibited the synthesis of PGs during postprandial hyperinsulinemia, which may be essential for regulating neuronal activity in the VMH. Inhibition of VMH neurons resulted in hyperphagia, possibly via POMC neurons in the ARC.

## Data Availability

Although we are not using an online data repository, the data that support the findings of this study are available from the corresponding author upon reasonable request.

## Abbreviations

The following abbreviations are used in this manuscript:

cPLA2	cytosolic phospholipase A2
SCRM	scrumble
VMH	ventromedial hypothalamus
PGs	Prostaglandins
CNS	central nervous system
KD	knockdown
COX	cyclooxygenase
dm	dorsomedial
ARC	arcuate nucleus
ERK	extracellular signal-regulated kinase
NTS	nucleus tractus solitarius
CCK	cholecystokinin
